# Fluorescence Polarization Screening Assays for Small Molecule Allosteric Modulators of ABL Kinase Function

**DOI:** 10.1371/journal.pone.0133590

**Published:** 2015-07-29

**Authors:** Prerna Grover, Haibin Shi, Matthew Baumgartner, Carlos J. Camacho, Thomas E. Smithgall

**Affiliations:** 1 Department of Microbiology and Molecular Genetics, University of Pittsburgh School of Medicine, Pittsburgh, Pennsylvania, United States of America; 2 Department of Computational and Systems Biology, University of Pittsburgh School of Medicine, Pittsburgh, Pennsylvania, United States of America; Hungarian Academy of Sciences, HUNGARY

## Abstract

The ABL protein-tyrosine kinase regulates intracellular signaling pathways controlling diverse cellular processes and contributes to several forms of cancer. The kinase activity of ABL is repressed by intramolecular interactions involving its regulatory Ncap, SH3 and SH2 domains. Small molecules that allosterically regulate ABL kinase activity through its non-catalytic domains may represent selective probes of ABL function. Here we report a screening assay for chemical modulators of ABL kinase activity that target the regulatory interaction of the SH3 domain with the SH2-kinase linker. This fluorescence polarization (FP) assay is based on a purified recombinant ABL protein consisting of the N-cap, SH3 and SH2 domains plus the SH2-kinase linker (N32L protein) and a short fluorescein-labeled probe peptide that binds to the SH3 domain. In assay development experiments, we found that the probe peptide binds to the recombinant ABL N32L protein in vitro, producing a robust FP signal that can be competed with an excess of unlabeled peptide. The FP signal is not observed with control N32L proteins bearing either an inactivating mutation in the SH3 domain or enhanced SH3:linker interaction. A pilot screen of 1200 FDA-approved drugs identified four compounds that specifically reduced the FP signal by at least three standard deviations from the untreated controls. Secondary assays showed that one of these hit compounds, the antithrombotic drug dipyridamole, enhances ABL kinase activity in vitro to a greater extent than the previously described ABL agonist, DPH. Docking studies predicted that this compound binds to a pocket formed at the interface of the SH3 domain and the linker, suggesting that it activates ABL by disrupting this regulatory interaction. These results show that screening assays based on the non-catalytic domains of ABL can identify allosteric small molecule regulators of kinase function, providing a new approach to selective drug discovery for this important kinase system.

## Introduction

The ABL protein-tyrosine kinase plays diverse roles in the regulation of cell proliferation, survival, adhesion, migration and the genotoxic stress response [1–3]. ABL kinase activity is perhaps best known in the context of BCR-ABL, the translocation gene product responsible for chronic myelogenous leukemia (CML) and some forms of acute lymphocytic leukemia [4,5]. The clinical management of CML has been revolutionized by selective ATP-competitive inhibitors of BCR-ABL, of which imatinib is the prototype [6]. However, chronic use of kinase inhibitors often leads to drug resistance due to selection for mutations that disrupt drug binding or allosterically influence the conformation of the drug binding pocket [7].

**Fig 1:**
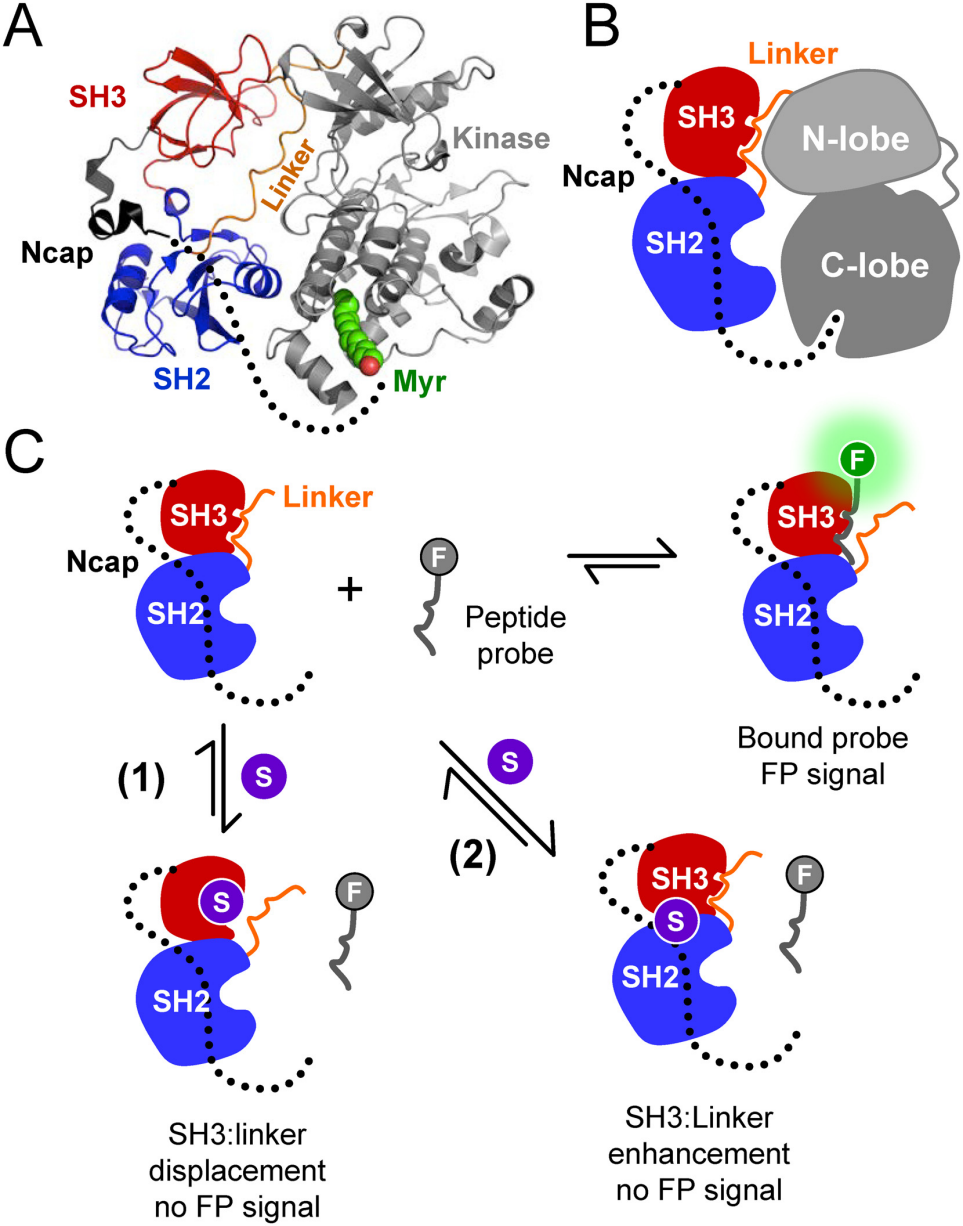
FP assay for small molecule modulators of ABL kinase function. A) Crystal structure of the auto-inhibited ABL core (PDB: 2FO0) [11]. Key features include the N-cap, SH3 and SH2 domains, the SH2-kinase linker, and the kinase domain. The disordered portion of the N-cap is indicated by the dotted line. The N-terminal portion of the N-cap is myristoylated and engages a deep pocket in the kinase domain C-lobe. B) Cartoon depiction of the intramolecular interactions regulating assembly of the downregulated ABL core. Note that the linker forms a polyproline helix that binds in *cis* to the SH3 domain. C) Fluorescence polarization (FP) assay. The FP assay combines a recombinant ABL Ncap-SH3-SH2-linker (N32L) protein and a SH3-binding peptide probe labeled with a fluorescent moiety (F). The probe peptide binds the SH3 domain in the ABL N32L protein, resulting in an FP signal. Small molecules (S) may bind to the SH3 domain and block probe peptide binding directly; such molecules would be expected to disrupt SH3:linker interaction (case 1). Alternatively, small molecules may stabilize SH3:linker interaction, making the SH3 domain inaccessible to the probe peptide (case 2). In either case, small molecule binding is predicted to result in a loss of the FP signal.

The growing problem of imatininb resistance in BCR-ABL has fueled efforts to identify compounds that work outside of the kinase active site. Such compounds offer advantages in terms of enhanced specificity, because they have the potential to exploit non-conserved regulatory features unique to ABL that persist to some extent in BCR-ABL as well [8]. The kinase activity of ABL is tightly regulated *in vivo* by an auto-inhibitory mechanism. The ABL ‘core’ region, which includes a myristoylated N-terminal ‘cap’ (N-cap), SH3 and SH2 domains, an SH2-kinase linker and the kinase domain, is both necessary and sufficient for ABL auto-inhibition [9]. Subsequent X-ray crystal structures of the ABL core revealed three critical intramolecular interactions that regulate kinase activity [10–12] (Fig 1A and 1B). First, the SH2-kinase linker forms a polyproline type II helix that binds to the SH3 domain, forming an interface between the SH3 domain and the N-lobe of the kinase domain. Second, the SH2 domain interacts with the back of the kinase domain C-lobe through an extensive network of hydrogen bonds. Aromatic interactions between the side chains of SH2 Tyr158 and kinase domain Tyr361 also help to stabilize this interaction (see Panjarian *et al*. for an explanation of the ABL amino acid numbering scheme [13]). Finally, the myristoylated N-cap binds a deep hydrophobic pocket in the C-lobe of the kinase domain, clamping the SH3 and SH2 domains against the back of the kinase domain. Small molecules that occupy the myristic acid binding site in the C-lobe of the kinase domain have proven to be effective allosteric inhibitors of BCR-ABL function [14,15].

Mutational analysis demonstrates that intramolecular SH3:linker interaction plays a central role in ABL auto-inhibition. Substitution of linker proline residues at positions 242 and 249 with glutamate disrupts SH3:linker interaction, resulting in ABL kinase activation [16]. In contrast, increasing the proline content of the linker enhances internal SH3 binding and overcomes the activating effects of mutations in the myristic acid binding pocket as well as the kinase domain gatekeeper residue (Thr315) [8]. Remarkably, enhanced SH3:linker interaction also dramatically sensitizes BCR-ABL-transformed cells to inhibition by both imatinib and the allosteric inhibitor, GNF-2, which binds to the myristic acid binding pocket [8]. These findings suggest that small molecules enhancing or disrupting this natural regulatory mechanism may represent selective allosteric modulators of ABL kinase activity.

In contrast to the tremendous research efforts invested in discovering inhibitors for ABL kinase activity, few studies have explored the discovery of small molecules that activate ABL. Selective agonists may represent useful probes to examine the role of ABL kinase activity in normal cellular functions, such as DNA-damage repair pathways. ABL is activated in response to multiple forms of genotoxic stress and interacts with modulators of both DNA damage- induced apoptosis (e.g. p73) and DNA repair (e.g. Rad51), resulting in cell death or survival depending on the cellular environment [2]. Moreover, recent studies have shown that ABL inhibits the growth of breast cancer xenografts and promotes the phenotypic reversion of invasive breast cancer cells [17,18]. Selective and potent agonists would provide a new approach to explore these and other biological roles of ABL in vivo.

In this study, we report the development and validation of a high-throughput screening assay for the identification of small molecules that interact directly with the non-catalytic region of the ABL core. Our assay is based on the interaction of a fluorescent probe peptide with the SH3 domain in the context of a recombinant protein encompassing the regulatory region of the ABL core (Ncap-SH3-SH2-linker; referred to hereafter as the ABL ‘N32L’ protein). Interaction of the probe peptide with the ABL N32L protein results in fluorescence polarization (FP), providing a convenient assay for SH3 occupancy in a format compatible with high-throughput chemical library screening. In theory, small molecules that bind to SH3 and disrupt probe binding may also disrupt SH3:linker interaction in the context of ABL, resulting in kinase activation. On the other hand, compounds that enhance internal SH3 binding to the natural linker may represent allosteric inhibitors. Using this FP approach, we screened a small library of FDA-approved drugs and identified a compound that specifically inhibited the FP signal from the complex of the ABL N32L protein with the probe peptide, suggesting that it may interfere with SH3:linker interaction. This compound, a substituted pyrimido-pyrimidine known as dipyridamole, was confirmed to bind directly to the ABL N32L protein using both differential scanning fluorimetry and surface plasmon resonance. Dipyridamole was found to enhance the activity of a recombinant downregulated ABL core protein, but had no effect on an ABL core with engineered high-affinity SH3:linker interaction or on the SRC-family kinase, HCK. These observations suggest that dipyridamole binds selectively to the ABL SH3 domain, resulting in linker displacement and kinase activation. This binding model is supported by molecular dynamics (MD) simulations and molecular docking. Our findings provide an important proof-of-concept that small molecules acting on the ABL SH3:linker interface can allosterically modulate ABL kinase activity, and provide a simple yet powerful assay method for their discovery.

## Materials and Methods

### Expression and purification of recombinant kinase proteins

The coding sequence for the ABL Ncap-SH3-SH2-linker region (N32L; corresponding to residues 2–255 with an internal deletion of residues 15–56; numbering based on the crystal structure of the human ABL core; PDB: 2FO0 [11]) was amplified by PCR and subcloned into the bacterial expression vector, pET21a (EMD Millipore). A similar construct was prepared using the sequence of ABL with a high-affinity linker (‘HAL9’) substitution as described previously [8]. One glycine and six histidine residues (GHHHHHH) were introduced at the N-terminus of the coding sequence of these proteins during sub-cloning. An inactivating mutation of the SH3 domain (W118A) was introduced by site-directed mutagenesis using the QuikChange II method (Stratagene) and the pET21a-ABL N32L WT plasmid as a template. The ABL N32L proteins were expressed in *E*.*coli* strain Rosetta2(DE3)pLysS (EMD Millipore) and purified using immobilized metal affinity chromatography. The purified proteins were then dialyzed against 20 mM Tris-HCl (pH 8.3) containing 200 mM NaCl and 1 mM DTT.

The wild type and high-affinity linker (‘HAL9’) ABL core proteins (residues 1–531 with an internal deletion of residues 15–56) were expressed in Sf9 insect cells as previously described [8]. The ABL core proteins were purified using a combination of ion-exchange and affinity chromatography and dialyzed against 20 mM Tris-HCl (pH 8.3) containing 100 mM NaCl and 3 mM DTT. The molecular weight and purity of all recombinant ABL proteins were confirmed by SDS-PAGE and mass spectrometry.

The SRC-family kinase HCK-YEEI was expressed in Sf9 insect cells and purified as previously described [19].

### Peptide synthesis

ABL SH3 domain-binding peptides p41, p40, p8, and 3BP-1 [20,21] were synthesized by the University of Pittsburgh Genomics and Proteomics Core Laboratories. For the FP assay, the peptides were labeled with 6-carboxyfluorescein at their N-termini. Molecular weight and purity of all peptides were verified by mass spectrometry. Stock solutions (10 mM) were prepared in a 1:1 mixture of DMSO and FP assay buffer (20 mM Tris-HCl, pH 8.3) for labeled peptides and neat FP assay buffer for unlabeled peptides. Peptide stock solutions were stored at -20°C.

### Fluorescence polarization assay

Fluorescence Polarization (FP) experiments were performed in quadruplicate in low volume black 384 well plates with a non-binding surface (Corning; catalog # 3676). Peptides and proteins were added to each well in FP assay buffer (20 mM Tris-HCl, pH 8.3) for a final assay volume of 20 μL and mixed by shaking for 5 min at room temperature. The FP signal in millipolarization (mP) units was measured at an excitation wavelength of 485 nM and emission wavelength of 515 nM in a SpectraMax M5 microplate reader (Molecular Devices) using the Softmax Pro software (version 5.4.1). Each plate was read three times and the values were averaged prior to analysis. Raw fluorescence intensity was also read at the same wavelengths for each assay.

### Chemical library screening

A collection of 1200 FDA-approved small molecules (Prestwick Chemical, Inc.) was used in a pilot screen with the FP assay. Each library compound was screened at 10 μM and a final DMSO concentration of 1%. Compounds were added to 384-well assay plates first, followed by a pre-mixed complex of the ABL N32L protein (25 μg) and the p41 probe peptide (50 nM). Each plate also contained twenty-eight wells of the wild type N32L protein plus p41 probe and DMSO as positive controls as well as twenty-eight wells of mutant ABL N32L-W118A protein plus p41 probe peptide and DMSO as negative controls. Each plate was mixed on the shaker for 5 min, read three consecutive times, and the average FP signal for each well was calculated. To identify potential hit compounds, three measures were used: (i) average FP signal; (ii) control normalized percent inhibition, in which the FP signal with each compound was normalized to the mean FP signal of the positive and negative plate controls according to the formula: [(sample FP–mean FP_WT_) / (mean FP_W118A_ –mean FP_WT_) x 100] [22,23]; (iii) Z score, a statistical measure of variation of the mean sample FP signal that is independent of plate controls, calculated according to the formula: [(sample FP–mean FP_samples_) / standard deviation_samples_] [22,23]. The compounds were then ranked in order of increasing FP signal, decreasing control normalized percent inhibition, and increasing Z score. Potential hit compounds were then retested in quadruplicate using the FP assay under screening assay conditions.

### Differential Scanning Fluorimetry

Hit compounds (100 μM) were pre-incubated with the ABL N32L WT protein (1 μM) for 30 minutes in bicine assay buffer (10 mM bicine, 150 mM NaCl, pH 8.0). SYPRO Orange (Sigma) was added at 5X final concentration and fluorimetry profiles were acquired with a StepOnePlus real-time quantitative PCR instrument (Applied Biosystems) and software (version 2.3). Assays were performed in duplicate in sealed MicroAmp Fast 96-well qPCR plates (Applied Biosystems), and control reactions without proteins were included to correct for background fluorescence. Assays were equilibrated at 25°C for 2 minutes, followed by an increase in temperature at the rate of 1% (1.6°C/min) to 99°C, with continuous data collection. Mean fluorescence intensities, after subtracting background fluorescence, were plotted against temperature. Non-linear regression analysis using the Boltzmann sigmoid function in GraphPad Prism 6 was used to determine the T_m_ values, the midpoint of the melt curve between the minimum and maximum fluorescence intensities.

### Surface Plasmon Resonance (SPR)

SPR analysis was performed on a BIAcore T100 instrument (GE Healthcare) using four-channel CM5 biosensor chips at 25°C. Recombinant purified ABL proteins were covalently attached to the CM5 chip via standard amine coupling chemistry [24,25]. Compound 142 (dipyridamole; Prestwick Chemical) was prepared in 20 mM Tris-HCl, pH 8.3, 150 mM NaCl and 0.1% DMSO and flowed past the immobilized ABL protein channel and a reference channel on the biosensor at a flow rate of 50 μL/min for 3 min over a range of concentrations. The initial binding reaction was followed by dissociation for 5 min, and the chip surface was regenerated using 20 mM Tris-HCl, pH 8.3, 150 mM NaCl, 0.1% DMSO, 0.05% Tween 20 and 1 mM DTT at a flow rate of 50 μL/min for 10 min. Sensorgrams were recorded in triplicate, corrected for buffer effects, and fitted with the 1:1 Langmuir binding model using the BIAevaluation software suite version 2.0.4 (GE Healthcare).

### Protein kinase assays

The ADP Quest assay (DiscoverRx) [26], which fluorimetrically measures kinase activity as the production of ADP, was used to determine ABL kinase reaction velocities. Assays were performed in quadruplicate in black 384 well plates (Corning #3571) in reaction volumes of 10 μL/well. Recombinant kinase protein concentrations were fixed at 40 ng/well for the wild type ABL core, 9 ng/well for the high affinity linker ABL core, and 250 ng/well for the SRC family kinase HCK. The Tyr2 substrate peptide for ABL (EAIYAAPFAKKK) as well as the SFK substrate peptide (YIYGSFK) were dissolved in the ADP Quest assay buffer (15 mM HEPES, pH 7.4, 20 mM NaCl, 1 mM EGTA, 0.02% Tween-20, 10 mM MgCl_2_, 0.1 mg/ml bovine γ-globulins), while ATP stocks were prepared in 10 mM Tris-HCl (pH 7.0). Each kinase reaction was initiated by the addition of ATP and read at 5 min intervals for 3 h in a SpectraMax M5 Microplate reader (Molecular Devices). To determine the substrate K_m_ for ABL kinases, the ATP concentration was fixed at 50 μM and the substrate peptide was serially diluted from 0.2–200 μM. For ABL kinase ATP K_m_ determination, the substrate concentration was fixed at the respective substrate K_m_ for each of the kinases, and the ATP concentration was titrated over the range of 0.2–200 μM. The resulting progress curves were analyzed according to the method of Moroco et al. [19]. Briefly, raw fluorescence data were corrected for non-enzymatic ADP production (no kinase or substrate control) and kinase auto-phosphorylation (rate observed in the absence of substrate), and converted to pmol ADP produced using an ADP standard curve generated under the same reaction conditions. The resulting values were normalized to the amount of kinase present in each reaction, and plotted against time. The linear portion of each progress curve was fit by regression analysis to determine the reaction velocity. Substrate and ATP K_m_ values were determined by non-linear regression analysis using the Michaelis-Menten equation (GraphPad Prism 6).

Half-maximal effective concentrations (EC_50_) and activation constants (K_act_) were determined for the ABL kinase core by both dipyridamole and the known ABL activator, DPH (5-(1,3-diaryl-1H-pyrazol-4-yl)hydantoin; Sigma-Aldrich) [27]. The kinase was pre-incubated with each compound (10 nM to 100 μM) for 30 min at room temperature, followed by kinase assay with the substrate and ATP concentrations fixed at their respective K_m_ values. The rate of each reaction was plotted against compound concentration, and analyzed by non-linear regression analysis (GraphPad Prism 6) to determine the EC_50_ value. To determine the activation constant K_act_, the basal rate of kinase activity was subtracted from the rates of reaction in the presence of each compound concentration, and plotted as a function of compound concentration. The resulting curves obeyed saturation kinetics and were best-fit by the following equation [19]:

Va= Vact[L](Kact+[L])

where V_a_ is the reaction velocity in the presence of each activator concentration, V_act_ is the maximal reaction velocity, L is the activator concentration, and K_act_ is the activator concentration that yields half-maximal reaction velocity.

### Molecular dynamics

To understand the dynamics of the recombinant N32L protein, for which there is no X-ray crystal structure, we ran unconstrained molecular dynamics (MD) simulations of residues 65–254 of the assembled, downregulated ABL core structure (PDB 2FO0). We disrupted the interaction between linker Pro249 and the SH3 domain by rotating the backbone bonds of linker Gly246. This glycine residue was chosen as a pivot point as it is more flexible and is located between Pro249 and the next strongly interacting residue (Val244) based on the predicted interaction energy with the SH3 domain [28]. We also ran MD simulations of the (unmodified) isolated SH3 domain (residues 80–145) using the same parameters described below.

MD simulations were conducted with the *pmemd*.*cuda* [29] module of AMBER14 [30], using the force fields AMBER ff14SB and gaff (general amber force fields) [31]. An octahedral TIP3P water box was constructed with 12 Å from the edge of the box to the solute and the total system charge was neutralized by adding chloride ions. The non-bonded cutoff was specified at 10 Å. In the first energy minimization run, the solute was held fixed and the solvent was relaxed through 500 cycles of steepest descent followed by 500 cycles of conjugate gradient minimization. Subsequently, the system was minimized again with no constraints through 2,000 cycles of steepest descent followed by 3,000 cycles of conjugate gradient minimization. Following the energy minimization, a 50,000 step MD simulation was used to raise the system temperature to 300 K while holding the solute fixed with weak (10.0 kcal/mol) restraints on the solute atoms. The bonds involving hydrogens were held at a fixed length and an integration step of 2 fs was used. This simulation was followed by a second equilibration simulation at constant pressure for 50,000 steps. The final MD simulation of this equilibrated structure was run with no constraints for 100 ns.

### Computational docking

The binding mode of hit compound 142 (dipyrimadole) was modeled to snapshots of the N32L and SH3 simulations by molecular docking using the program smina [32] with default docking parameters. The box was defined by the coordinates of linker residues 247–251 plus an outer shell of 8 Å after alignment to the SH3 domain of the crystal structure (PDB 2FO0).

### Transient expression of ABL core proteins in 293T cells

ABL core proteins were expressed in human 293T cells and analyzed for kinase activity as described elsewhere [8]. Briefly, cells were grown overnight in 60 mm plates, transfected with an expression vector for the wild type ABL core, and treated with compounds 2 h post- transfection. Cells were lysed by sonication 24 h later, and cell lysates clarified by centrifugation. ABL was immunoprecipitated via the C-terminal His-tag, resolved by SDS-PAGE, and transferred to nitrocellulose membranes for immunoblot analysis with antibodies to the ABL protein and the three autophosphorylation sites: pTyr412 (activation loop), pTyr245 (SH2-kinase linker), and pTyr89 (SH3 domain).

## Results and Discussion

### ABL fluorescence polarization (FP) assay design

In this study, we developed a screening assay for small molecule allosteric modulators of ABL kinase function. Our goal was to enable discovery of chemical scaffolds that interact with the regulatory region of the ABL kinase core, as opposed to the kinase domain, thereby providing a path to enhanced selectivity and allosteric control of kinase function. In addition, we wanted a flexible assay with the potential to identify both inhibitors and activators of ABL function. To accomplish these goals, we developed a fluorescence polarization (FP) assay based on the N-terminal region of ABL consisting of the Ncap, SH3 and SH2 domains, and the SH2-kinase linker (ABL N32L protein). Binding of a fluorescently labeled probe peptide to the SH3 domain (displacing the linker) should result in an increased FP signal due to the slowed rotation of the N32L target protein-peptide complex (Fig 1C). A small molecule that binds to the ABL N32L protein and enhances SH3 interaction with the linker in *cis* is predicted to prevent probe peptide binding, resulting in a decrease in the FP signal. Molecules in this class are predicted to act as allosteric inhibitors of ABL kinase activity, because they may enhance the natural negative regulatory interaction between the SH3 domain and the linker. Alternatively, compounds that interact with the SH3 domain and block probe peptide binding are also predicted to cause a decrease in the FP signal. By displacing SH3:linker interaction in the context of downregulated ABL, compounds of this type may act as allosteric activators of kinase activity. This assay design therefore has the potential to identify both types of ABL-binding compounds in a single chemical library screen. Their impact on ABL function can be easily distinguished in secondary assays for direct binding to the ABL domains, as well as functional assays.

### Recombinant ABL regulatory proteins for FP assay development

**Fig 2:**
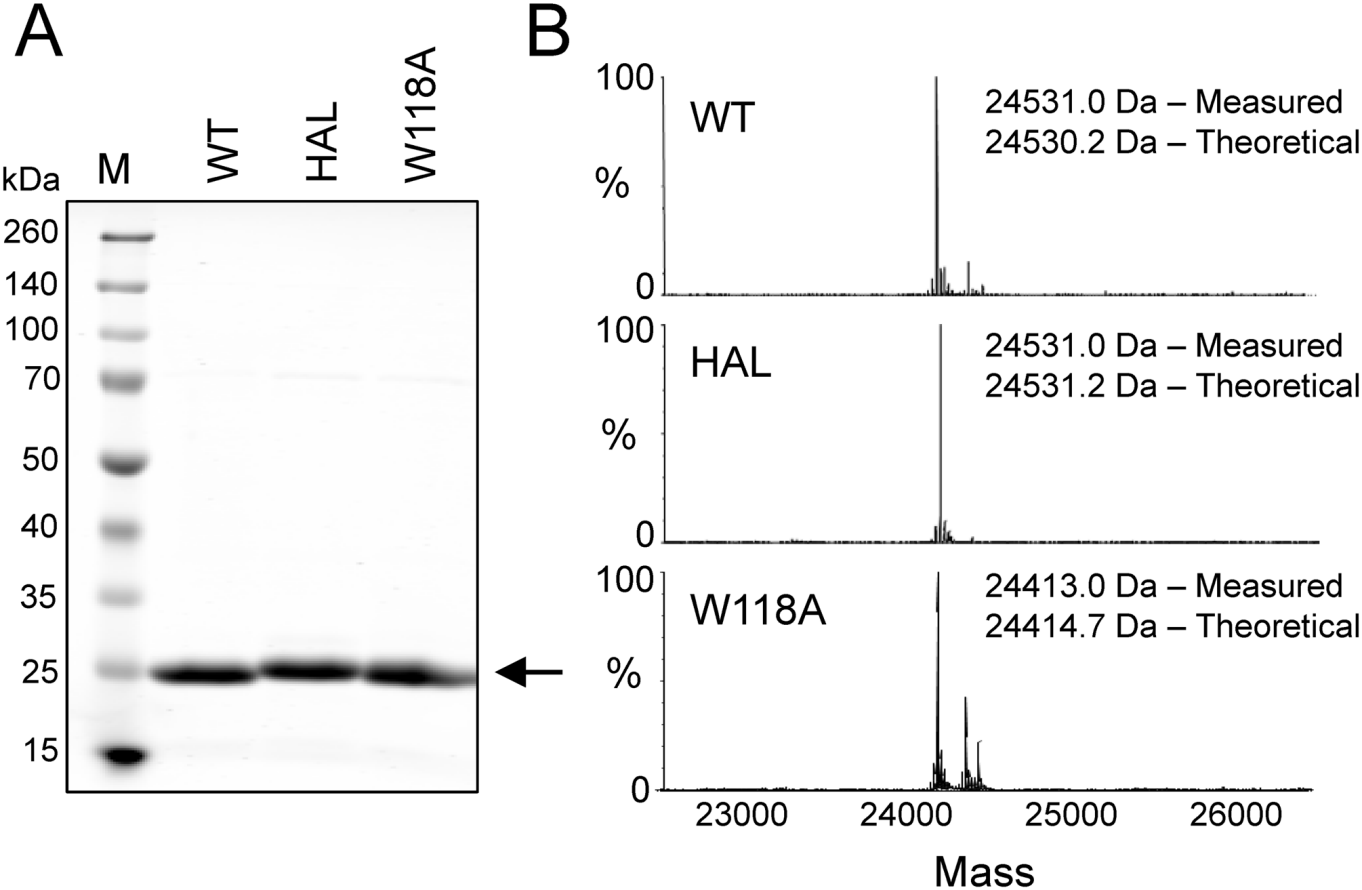
Recombinant ABL Ncap-SH3-SH2-linker (N32L) proteins. Wild type ABL N32L protein and the corresponding high-affinity linker (HAL) and W118A mutants were expressed in *E*. *coli* using the pET system [40] and purified by immobilized metal affinity chromatography. Protein purity and mass were verified by SDS polyacrylamide gel electrophoresis (A) and mass spectrometry (B).

**Fig 3:**
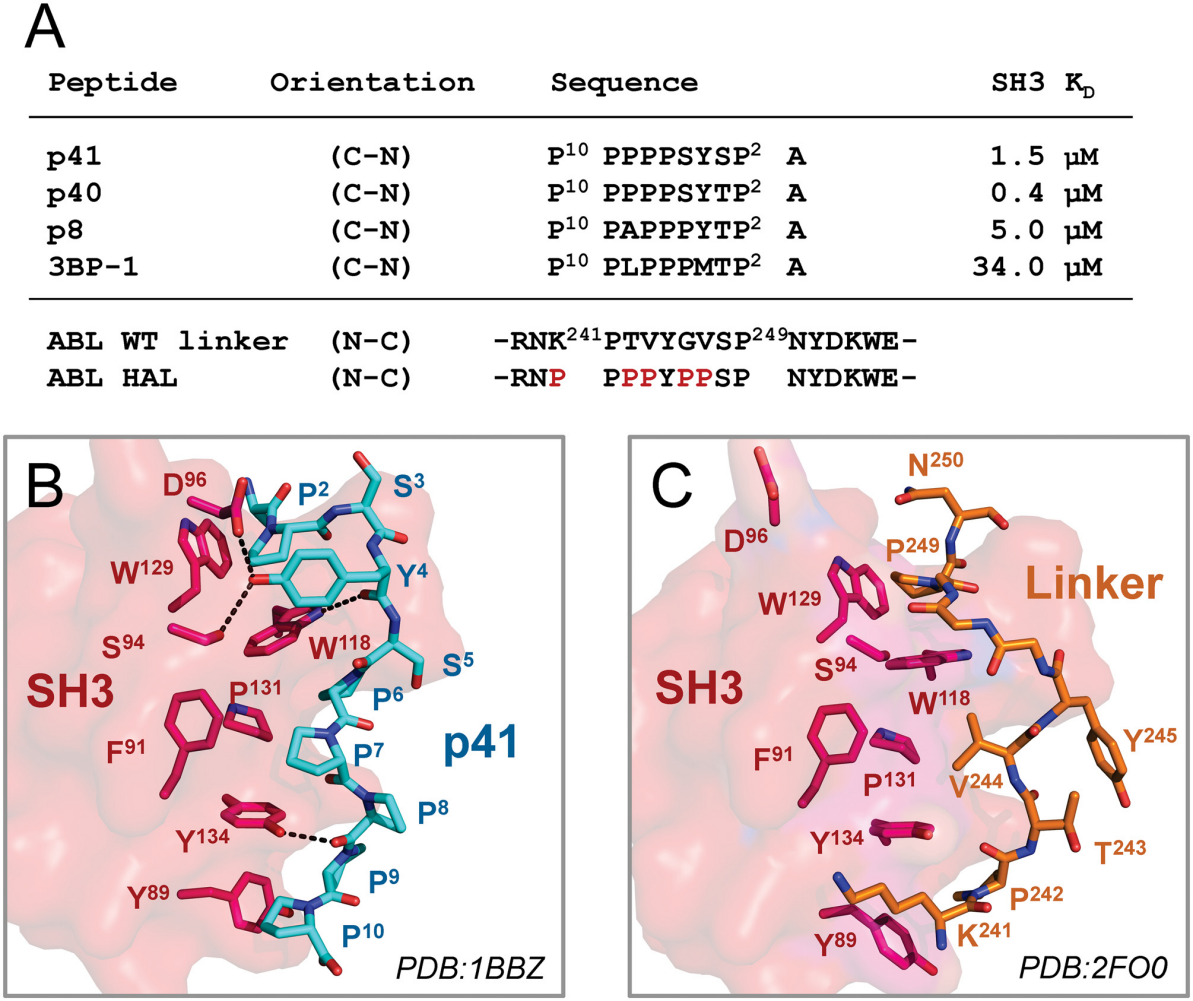
Peptide and linker interactions with the ABL SH3 domain. A) Sequences of the ABL SH3 binding peptides, p41, p40, p8, and 3BP-1, and their published binding affinities for the ABL SH3 domain [20,21]. Sequences of the wild type (WT) and high-affinity (HAL) SH2-kinase linker sequences are also shown at the bottom. The peptide sequences are presented in the C- to N-terminal orientation to align with those of the linkers. B) Crystal structure of the p41 peptide (cyan) bound to the ABL SH3 domain (PDB: 1BBZ) [21]. The SH3 surface is shown as a space filling model (red) and side chains of residues that interact with the p41 peptide are shown as sticks. C) Crystal structure of the SH2-kinase linker (orange) bound to the ABL SH3 domain (red) from the ABL core (PDB: 2FO0) [11]. Side chains of SH3 domain residues that interact with the p41 peptide as per panel B are shown as sticks. Note the lack of hydrophobic interactions and hydrogen bonds between the SH3 domain and the linker in comparison to the p41 peptide.

The target protein for the ABL FP assay consists of the first 255 residues of ABL (isoform 1b), and encompasses the Ncap, the SH3 and SH2 domains, as well as the SH2-kinase linker as described above. This ABL N32L protein was expressed in bacteria in soluble form, purified to homogeneity, and its purity and identity were confirmed by SDS-polyacrylamide gel electrophoresis and mass spectrometry, respectively (Fig 2). Previous studies have established that regulatory SH3:linker interaction is maintained in this construct, despite the absence of the kinase domain [8,33]. In addition to the wild type protein, two mutant forms of N32L were produced for use as controls. The first of these has an alanine substitution for a conserved tryptophan on the SH3 domain binding surface (W118A mutant; see Fig 3 for SH3 domain structure), which renders it unable to bind to the probe peptide and thus serves as a negative control. In the second mutant, five linker residues were replaced with prolines to enhance interaction with the SH3 domain [8]. This high-affinity linker (HAL) substitution suppresses the activating effects of kinase domain mutations and influences the conformation of the kinase domain, enhancing both imatinib and allosteric inhibitor action (see Introduction). The HAL protein therefore represents a second negative control for probe peptide binding to the SH3 domain. Both the W118A and HAL forms of the ABL N32L protein were also expressed and purified from bacteria, and yielded soluble purified proteins of the expected mass (Fig 2).

### Structural basis for high affinity probe peptide binding to the ABL SH3 domain

A suitable probe for the ABL N32L FP assay required a short, proline-rich peptide with sequence specificity for the ABL SH3 domain. In addition, the probe peptide needed to bind to the SH3 domain with sufficient affinity to compete for *cis*-interaction of the SH3 domain with the natural linker (Fig 1). A survey of the literature identified four ABL SH3-binding peptides with the potential to serve as probes [20,21]. These peptides, designated p41, p40, p8, and 3BP-1, have K_D_ values for the ABL SH3 domain in the 0.4 to 34 μM range. The ABL SH3-binding peptide sequences are presented in Fig 3A, and are aligned with those of the wild type and high-affinity SH2-kinase linkers of ABL.

To explore the potential of known ABL SH3 peptide ligands to compete for natural SH3:linker interaction, we first compared the structure of the ABL SH3:linker interface from the downregulated ABL core (PDB: 2FO0) [11] with the crystal structure of the p41 peptide in complex with the ABL SH3 domain (PDB: 1BBZ) [21]. The C-terminal half of the p41 peptide is comprised exclusively of proline, which facilitates both PPII helix formation as well as tight interaction with the hydrophobic SH3 binding surface (Fig 3B). In contrast, this region of the SH2-kinase linker is comprised of the less favorable SH3-binding sequence, KPTVY (Fig 3C). Specifically, p41 proline residues 9 and 10 fill the hydrophobic groove formed by the aromatic side chains of SH3 tyrosines 89 and 134; the linker is substituted with lysine in this position (Lys241). The main chain carbonyl of p41 Pro8 forms a stabilizing hydrogen bond with Tyr134. This position is substituted with threonine (Thr243) in the linker, which swings away from the SH3 surface. The N-terminal sequence of the p41 peptide forms a network of polar contacts involving SH3 residues Ser94, Asp96, and Trp118. None of these contacts are present in the SH3:linker interface, and the side chain of SH3 Asp96 is rotated away from the linker. Taken together, these structural features strongly suggested that p41, or one of the closely related peptides (p40, p8, and 3BP-1), may interact with the ABL N32L target protein with sufficient affinity to displace the wild type linker and provide a stable FP signal.

### Selection of a probe peptide for the ABL N32L FP assay

**Fig 4:**
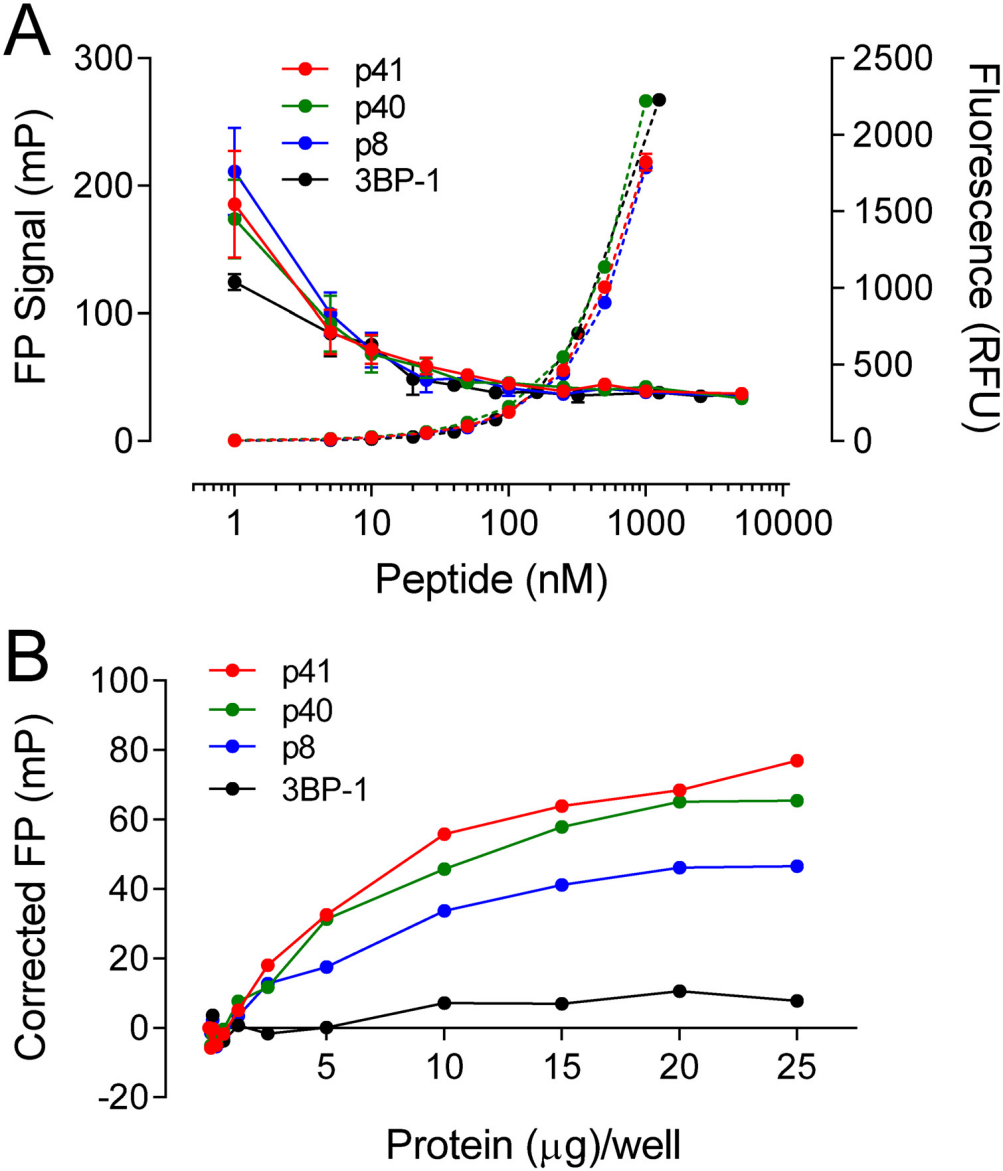
Identification of p41 as optimal probe peptide for the ABL N32L FP assay. A) To characterize the baseline FP signal, the 6-carboxy-fluorescein labeled probe peptides p41 (red), p40 (green), p8 (blue), and 3BP-1 (black) were serially diluted in the concentration range of 1–1000 nM. The FP signals (solid lines, left Y axis) and corresponding fluorescence intensities (dashed lines, right Y axis) were measured and plotted as a function of peptide concentration. Average values are shown ± SE from four measurements per condition. B) To test for probe peptide interaction with ABL N32L by FP, each peptide (50 nM) was incubated with the ABL N32L protein over the range of 0.08–25 μg/well. The resulting FP signals were corrected for baseline FP signal recorded in the absence of the N32L protein and plotted against the N32L protein concentration. Average FP values are shown ± SE from four measurements per condition; error bars are smaller than the diameter of some data points.

To evaluate the suitability of the four ABL SH3 peptide ligands (p41, p40, p8, 3BP-1; Fig 3A) as FP probes, each peptide was synthesized and labeled with 6-carboxyfluorescein on its N-terminus. We first examined the baseline FP signal as well as the fluorescence intensity exhibited by each labeled peptide over a broad concentration range (1–1,000 nM) in the absence of the ABL N32L target protein. As shown in Fig 4A, probe peptide concentrations greater than 50 nM exhibited stable baseline FP readings with minimal well-to-well variation.

To test for ABL N32L protein interaction with each peptide in the FP assay, we held each probe peptide concentration at 50 nM and added the wild type N32L protein over a range of concentrations. As shown in Fig 4B, both the p40 and p41 probe peptides produced a strong, saturable FP signal as a function of the N32L protein concentration. The p8 peptide also produced an FP response, albeit somewhat lower than that observed with p40 and p41, while the 3BP-1 peptide was inactive. These FP results correspond to the rank order of binding affinities previously reported for these peptides with the isolated SH3 domain [20,21]. Since the structure of the ABL SH3 domain in complex with p41 is known (Fig 3B), we chose the p41 peptide for FP assay optimization.

### ABL N32L FP assay development and optimization

**Fig 5:**
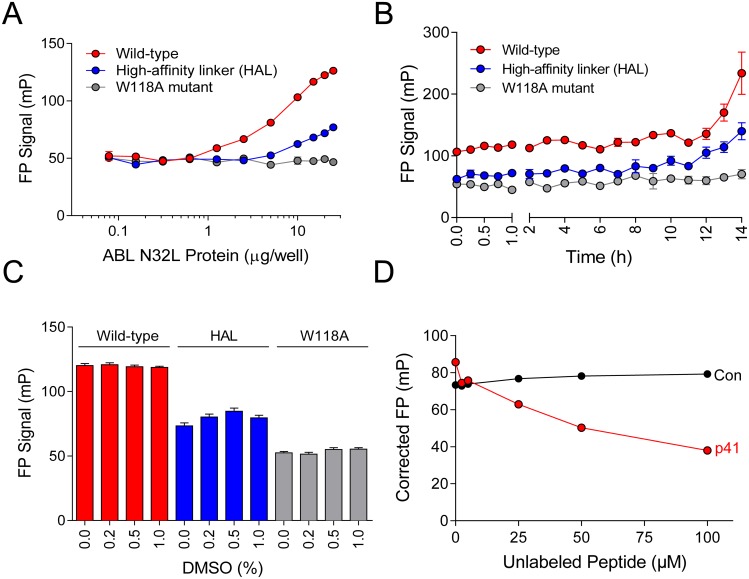
ABL N32L FP assay development and optimization. A) *The p41 FP probe binds the ABL N32L protein through the SH3 domain*. The p41 probe peptide (50 nM) was combined with wild type, HAL, and W118A ABL N32L proteins over the range of concentrations shown. The resulting FP signals were measured and plotted as a function of N32L protein concentration. B) *FP assay stability*. The p41 probe peptide (50 nM) was combined with the three ABL N32L proteins (12.8 μg/well) and FP signals were recorded over the time course shown. C) *DMSO tolerance*. FP assays consisting of the p41 probe peptide (50 nM) and each ABL N32L protein (25 μg/well) were incubated with the DMSO concentrations shown, and FP signals were recorded 1 h later. D) *Unlabeled peptide competition*. For the competition assay, the p41 probe peptide (50 nM) was mixed with unlabeled p41 peptide or a negative control peptide of unrelated sequence (QKEGERALPSIP) and similar length (Con) over the range of concentrations shown. The ABL N32L protein (20 μg/well) was then added, and FP signals were recorded. FP signals were corrected for the background p41 peptide FP signal and plotted as a function of the unlabeled peptide concentration. In all experiments (A through D), average FP values are shown ± SE from four measurements per condition.

We next investigated whether the FP signal obtained with the p41 probe peptide was due to interaction with SH3 domain of the recombinant ABL N32L target protein. For these experiments, we compared the FP signal produced from the wild type ABL N32L protein with the SH3 domain mutant (W118A) as well as the high-affinity linker (HAL) protein. As shown in Fig 5A, the wild type ABL N32L protein produced a concentration-dependent increase in the FP signal as observed previously. In contrast, the N32L W118A mutant failed to produce an FP signal with the p41 peptide over the same concentration range, indicating that the peptide requires this conserved SH3 domain tryptophan residue for binding as predicted from the crystal structure (see Fig 3). On the other hand, the ABL N32L HAL protein showed a greatly reduced FP signal in comparison to the wild type protein with the p41 probe. This result is consistent with enhanced *cis*-interaction of the linker with SH3 domain in this protein as a result of the higher linker proline content (see Fig 3A for HAL sequence). Results with these control proteins demonstrate that the p41 probe peptide interacts with the ABL N32L target protein through its SH3 domain. FP experiments with the recombinant purified ABL SH3 domain alone also produced a very similar FP response, supporting this conclusion (data not shown). Findings with these ABL N32L mutants support the idea that small molecules that disrupt or stabilize intramolecular interaction between the SH3 domain and linker will also reduce probe peptide binding and loss of the FP signal.

We next tested the stability of the FP signal as a function of time (Fig 5B). For this experiment, the p41 probe peptide (50 nM) and ABL N32L protein (12.8 μg/well) concentrations were held constant. Under these conditions, no significant variation in the FP signal was observed up to 10 hours. We also found that DMSO, the carrier solvent for the screening library compounds, did not influence the FP signal or the negative controls even at the highest concentration tested (1%; Fig 5C).

In a final validation experiment, we tested the effect of unlabeled p41 peptide on the FP signal (Fig 5D). For this study, we fixed the p41 probe peptide concentration at 50 nM and the wild type ABL N32L protein concentration at 20 μg/well. Unlabeled p41 peptide was added to the assay over the concentration range of 2.5 to 100 μM. The FP signal decreased as a function of unlabeled p41 peptide concentration, demonstrating competition for the labeled probe peptide binding to the N32L protein. As a negative control, the peptide competition experiment was repeated with a non-specific peptide of similar length. This peptide had no effect on the FP signal, even at a concentration of 100 μM, demonstrating the specificity of p41 peptide recognition by the SH3 domain in the N32L target protein.

### Identification of inhibitors of p41 interaction with ABL N32L

**Fig 6:**
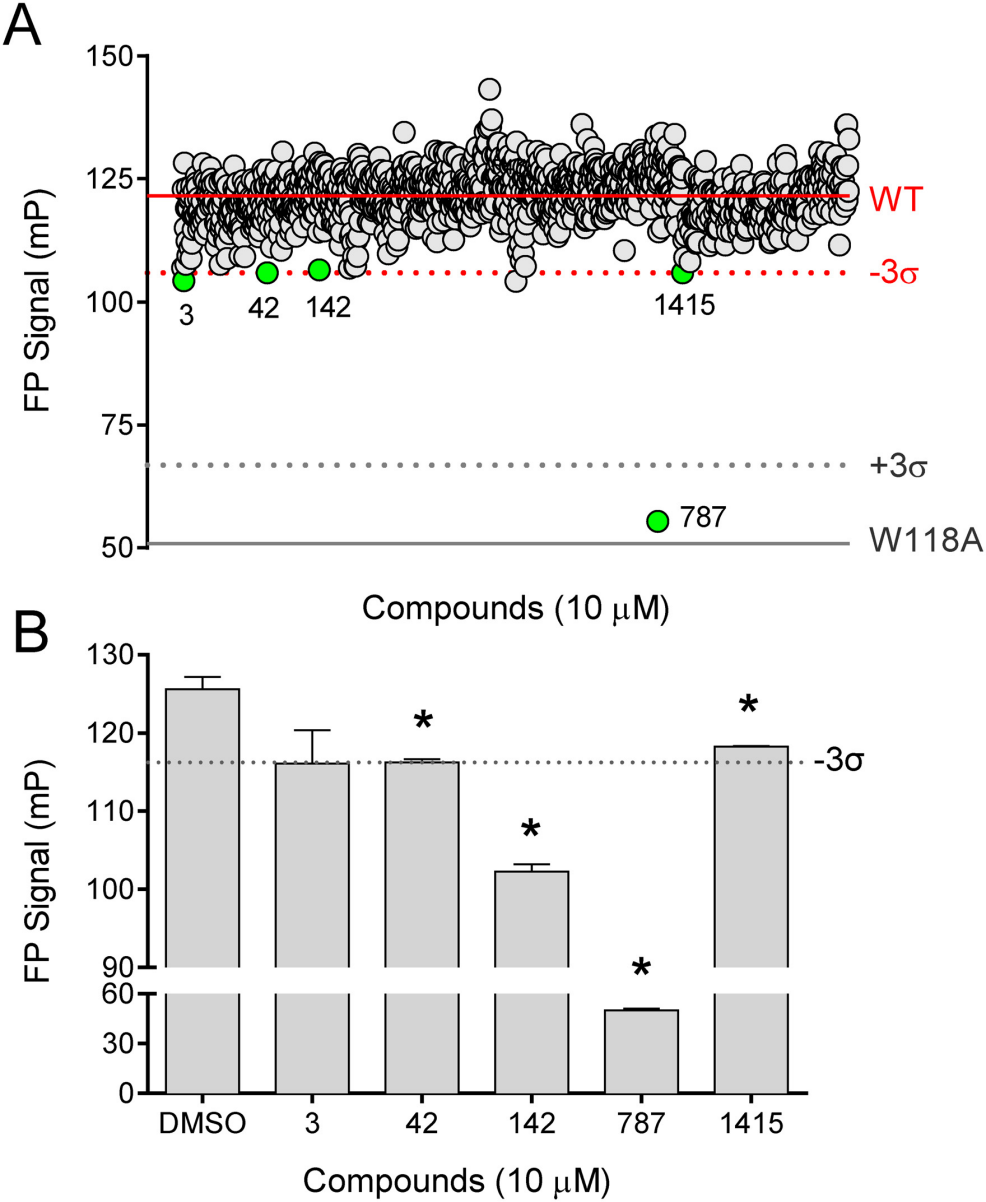
Pilot screen identifies inhibitors of p41 interaction with the ABL N32L protein. A) A library of 1200 FDA-approved compounds was screened using the ABL N32L protein (25 μg/well) and the p41 probe peptide (50 nM) in the FP assay. The solid lines correspond to the mean FP signals for the wild type (WT) and SH3 mutant (W118A) control N32L proteins across all assay plates, with the dotted lines indicating three standard deviations from the means (± 3σ). Compounds were screened at 10 μM and each FP signal is represented as an individual circle. Five putative hit compounds were identified (green circles). B) The five potential hit compounds were re-tested in quadruplicate at 10 μM vs. the DMSO control under FP screening assay conditions, and the mean FP values are shown ± SE. Four of these compounds significantly inhibited the FP signal relative to the DMSO control as indicated by the asterisk (p < 0.05; 2-tailed t-test). The dotted line shows the FP value three standard deviations below the DMSO control FP signal (-3σ).

To test the performance of the ABL N32L FP assay under screening conditions, we performed a pilot screen of 1200 FDA-approved compounds. The wild type ABL N32L protein (25 μg) was added to each well together with the p41 probe peptide (50 nM). The compounds were then added to a final concentration of 10 μM in 1% DMSO. Each plate contained twenty-eight wells with the wild type N32L target protein plus DMSO as positive controls, and twenty-eight wells with the non-binding W118A mutant protein plus DMSO as negative controls. The overall Z factor for the pilot screen was 0.57, indicative of a reliable screening assay [34]. The average FP signals observed with the controls as well as the readings observed with each of the test compounds are presented in Fig 6A.

The FP signals for each compound were ranked by three different methods: increasing raw FP signal, decreasing normalized percent inhibition, and increasing Z score (see Materials and Methods). We then compared the top 1% of compounds present in each of these three rankings. Five compounds were present in at least two of these rankings, and were selected for follow-up assays (compound numbers 3, 42, 142, 787, and 1415; Fig 6A). Each of the raw hit compounds was then retested in multiple wells under screening assay conditions (Fig 6B). Four out of the five compounds produced a significant inhibition of the FP signal relative to the DMSO controls (compounds 42, 142, 787, and 1415). We then performed control FP experiments with each of these compounds under the same conditions but in the absence of the N32L target protein. This counter-screen showed that compound 787 also inhibited the baseline FP signal produced by the p41 probe peptide, indicating non-specific quenching of the FP signal (data not shown). None of the other compounds affected baseline p41 probe peptide fluorescence, and were therefore moved forward into secondary assays.

### Compounds identified in the ABL N32L FP screen interact directly with the ABL N32L protein in orthogonal assays

**Fig 7:**
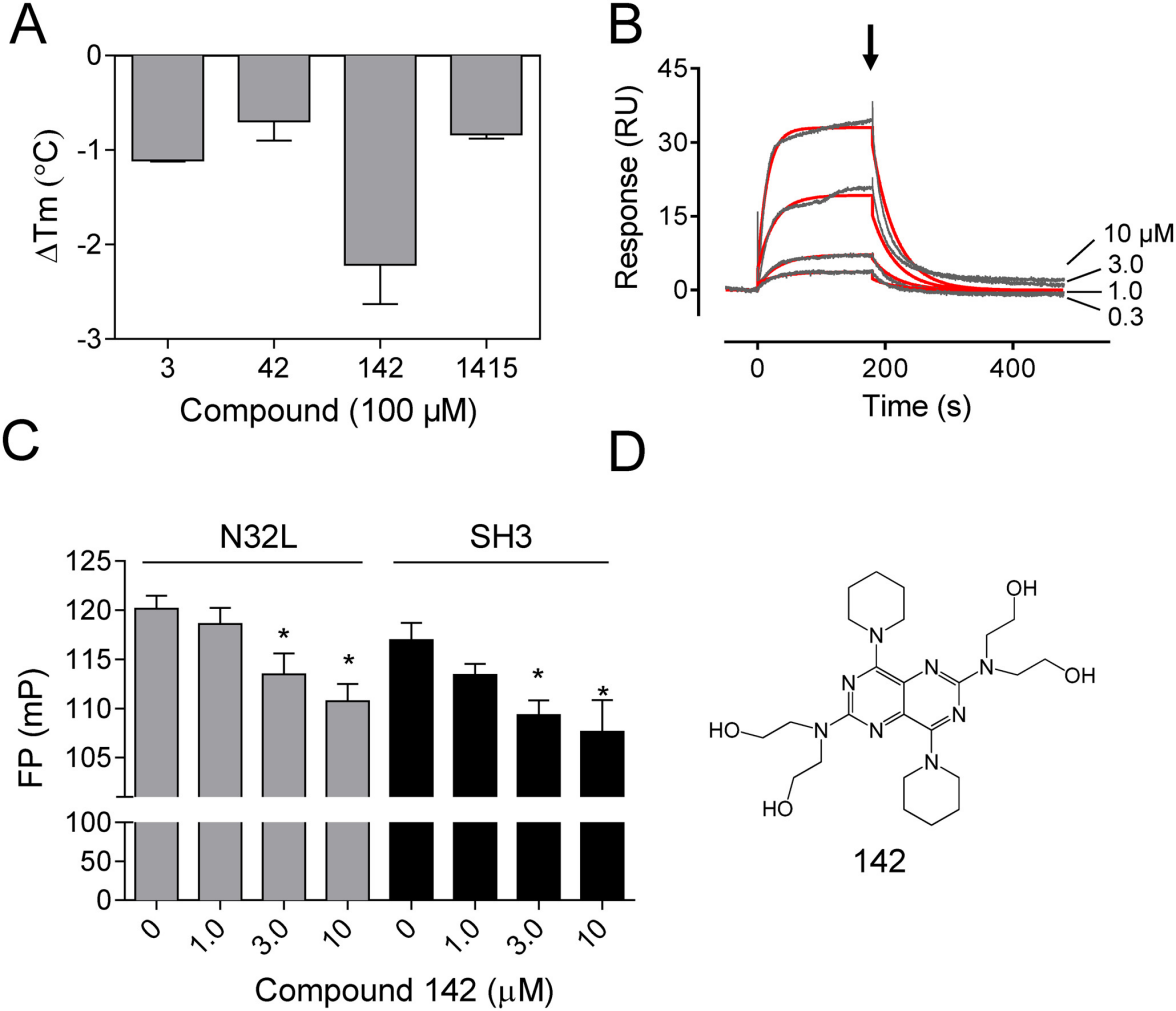
Hit compound 142 interacts directly with the ABL N32L protein. A) Differential scanning fluorimetry assays were performed on the ABL N32L protein in the presence of the four confirmed hit compounds as described under Materials and Methods. The average change in the mid-point of the thermal melt profile (ΔT_m_) relative to the T_m_ obtained with the N32L protein in the presence of the DMSO carrier solvent is plotted on the Y-axis ± SE (n = 2). B) Surface plasmon resonance was performed with the ABL N32L protein immobilized on the biosensor chip and compound 142 as analyte. Responses were recorded for the four compound concentrations shown, and the flow path was switched back to buffer after 180 s to induce dissociation (*arrow*). The resulting sensorgrams (black lines) were fit by a 1:1 Langmuir binding model (red lines) to generate kinetic constants. C) Compound 142 inhibits p41 peptide binding to the ABL N32L and SH3 proteins in the FP assay. Compound 142 was added to N32L and SH3 FP assays over the range of concentrations shown, and the resulting FP signals are presented as the mean ± SE. Significant inhibition for both N32L and SH3 was observed at 3 and 10 μM (*p < 0.05 by 2-tailed t-test). D) Chemical structure of compound 142 (dipyridamole).

As an independent measure of hit compound interaction with the ABL N32L target protein, we performed differential scanning fluorimetry assays [35]. For these experiments, the ABL N32L protein was heated with a molar excess of each compound in the presence of the reporter dye, SYPRO orange. As the temperature rises and the N32L protein unfolds, the reporter dye accesses the hydrophobic interior of the protein, resulting in an increase in dye fluorescence. The resulting protein ‘melt curve’ is then fit by regression analysis to obtain a T_m_ value, the temperature at which half-maximal thermal denaturation is observed. Small molecule binding to a target protein can either increase or decrease the T_m_ value, depending upon the effect of the compound on protein stability. Differential scanning fluorimetry was performed with the wild type N32L protein in the presence of each of the hit compounds from the FP assay, and the change in T_m_ value (ΔT_m_) was determined compared to DMSO as the reference control. As shown in Fig 7A, all four compounds produced a significant decrease in the T_m_ value. Compound 142 had the largest impact on N32L thermal stability, producing a decrease of more than 2°C in the T_m_, consistent with its effect in the FP assay (Fig 6B).

To confirm direct interaction of compound 142 with the ABL N32L protein and explore the binding kinetics, we next performed surface plasmon resonance assays. For these experiments, the ABL N32L protein was immobilized on the biosensor surface while compound 142 was flowed past the immobilized protein over a range of concentrations. As shown in Fig 7B, concentration-dependent interaction of compound 142 with ABL N32L was readily detected by this approach, yielding an association rate constant of 3.87 ± 0.59 x 10^3^ M^-1^s^-1^ and a dissociation rate constant of 2.70 ± 0.72 x 10^−2^ s^-1^. The equilibrium dissociation constant (K_D_) for this interaction, calculated as the ratio of k_d_/k_a_, is 6.90 ± 0.78 x 10^−6^ M.

Compounds that inhibit the FP signal in the ABL N32L assay may either interfere directly with probe peptide binding to the SH3 domain or allosterically tighten the cis-interaction of the SH3 with the linker, indirectly reducing probe peptide interaction. To distinguish between these two possibilities with compound 142, we performed FP assays with the N32L protein as well as the isolated ABL SH3 domain. As shown in Fig 7C, Compound 142 resulted in a concentration-dependent decrease in the FP signal with both the ABL N32L and SH3 proteins, suggesting that this compound binds directly to the ABL SH3 domain.

### Allosteric activation of ABL kinase by compound 142

Compound 142 reproducibly scored as a hit in the ABL N32L FP assay and demonstrated direct interaction with ABL N32L protein by both differential scanning fluorimetry and SPR. This compound, a symmetrically substituted pyrimido-pyrimidine known as dipyridamole (Fig 7D), is a selective inhibitor of phosphodiesterase V and also an adenosine transport inhibitor used clinically for its antithrombotic activity [36]. However its potential impact on protein kinase function has not been reported. Because compound 142 interacts with the regulatory region of ABL, we investigated its effects on ABL kinase activity using two purified recombinant forms of ABL in a kinetic kinase assay. These included the wild type ABL core region, consisting of the Ncap, the SH3 and SH2 domains, the SH2-kinase linker, and the kinase domain. This ABL protein was produced in Sf9 insect cells, which results in myristoylation of the N-cap and interaction with the C-lobe of the kinase domain, thereby assembling the downregulated state (Fig 1A). In addition to the wild type core, we also tested a high-affinity linker (HAL) mutant version of the ABL protein, which has a modified proline-rich linker that packs more tightly against the SH3 domain [8].

Baseline kinase activities and kinetic parameters of each recombinant ABL kinase core protein were determined first using a fluorimetric assay that measures the kinase reaction rate as the generation of ADP [26]. For wild type ABL, we obtained K_m_ values of 9.78 ± 0.14 μM and 144.65 ± 1.64 μM for ATP and peptide substrate, respectively. The ABL HAL core yielded a similar K_m_ value for substrate (150.25 ± 5.35 μM), with a higher value for ATP (21.24 ± 1.6 μM). In subsequent experiments, the ATP and peptide substrate concentrations were set to their respective K_m_ values, and input kinase concentrations were adjusted to yield the same basal reaction rates (7 pmol ADP produced per minute).

**Fig 8:**
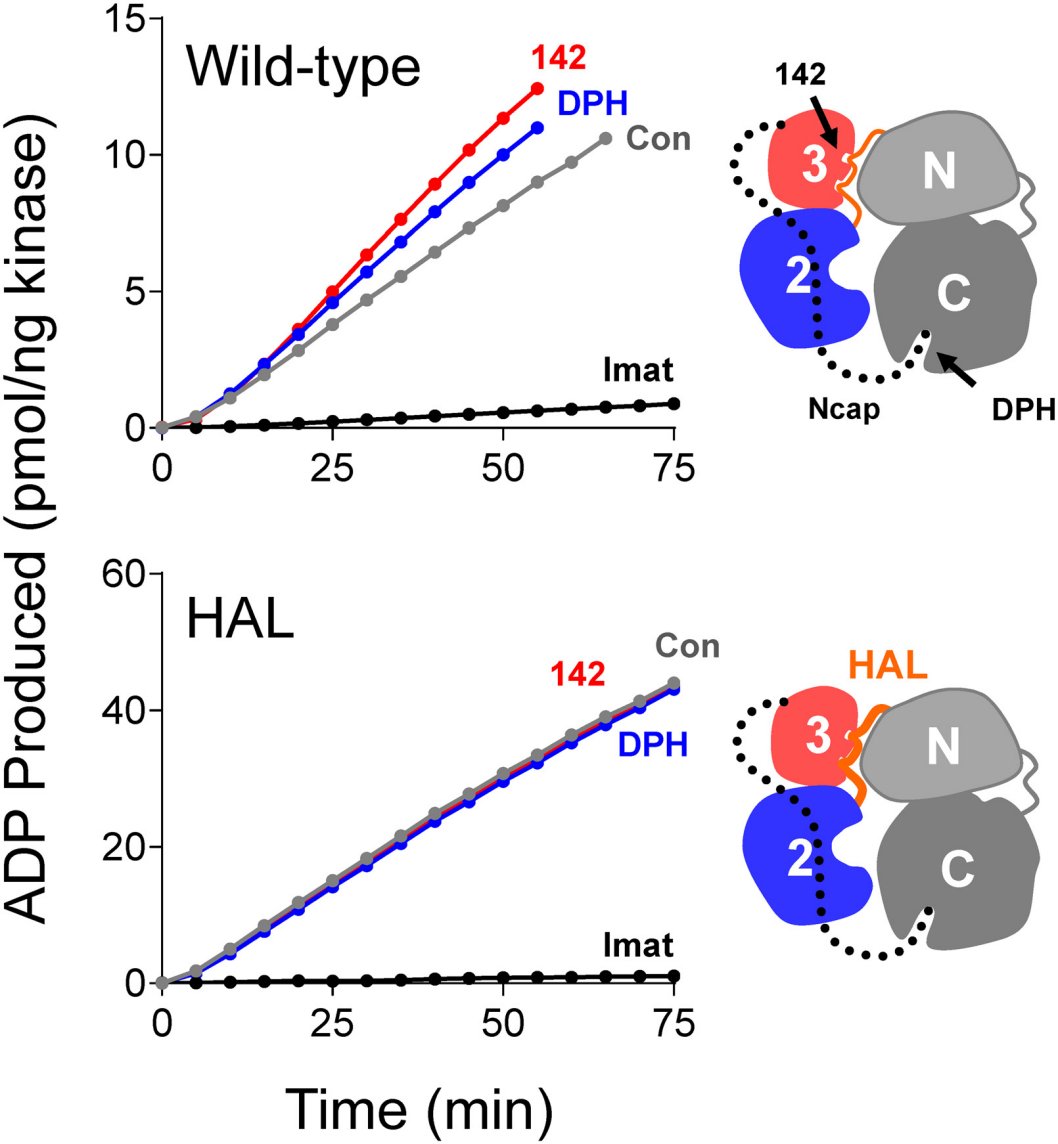
Compound 142 activates the ABL kinase core in vitro. *Top*: The recombinant ABL core protein, consisting of the Ncap, SH3, SH2 and kinase domains, was assayed in the presence of compound 142 (10 μM), the known ABL activator DPH (10 μM), and imatinib (1 μM) or with DMSO as control (Con) using a kinetic kinase assay (see Materials and Methods). Data are plotted as pmol ADP produced per ng kinase as a function of time. The cartoon (right) depicts the domain organization of the wild type ABL core, and indicates the binding site for DPH (myristic acid binding pocket) as well as the predicted binding site for compound 142 (SH3 domain). *Bottom*: Kinase assays were performed using an ABL core protein with a high-affinity linker (HAL) in the presence of the same three compounds; the cartoon indicates the position of the modified linker (HAL). In both cases, the ATP and peptide substrate concentrations were set to their respective K_m_ values (wild type ABL: 9.78 ± 0.14 μM for ATP and 144.65 ± 1.64 μM for substrate; ABL HAL: 21.24 ± 1.6 μM for ATP and 150.25 ± 5.35 μM for substrate).

We first examined the effect of compound 142 on the activity of the wild type ABL kinase core protein. As shown in Fig 8, compound 142 stimulated wild type ABL kinase activity by about 40% at a concentration of 10 μM relative to the DMSO control in this assay. As a positive control, we also assayed ABL core activity in the presence of the same concentration of a previously described ABL activator, DPH, and observed a similar degree of activation. DPH, unlike compound 142, stimulates ABL through the kinase domain via the myristic acid binding pocket in the C-lobe [27].

The mechanism of ABL activation by compound 142 may involve binding to the SH3 domain and subsequent displacement of its regulatory interaction with the SH2-kinase linker. Indeed, mutations that disrupt SH3:linker interaction also have a stimulatory effect on ABL kinase activity (see Introduction). To test this idea, we next examined the effect of this compound on the ABL core mutant with enhanced SH3:linker interaction. Unlike wild type ABL, compound 142 did not affect the kinase activity of the ABL core with the HAL substitution (Fig 8), consistent with the idea that enhanced SH3:linker interaction prevents compound 142 access to the ABL SH3 domain. Interestingly, DPH did not activate the ABL HAL core protein either, consistent with previous results showing that enhanced SH3:linker interaction overcomes ABL core activation by mutations in the myristic-acid binding pocket [8].

**Fig 9:**
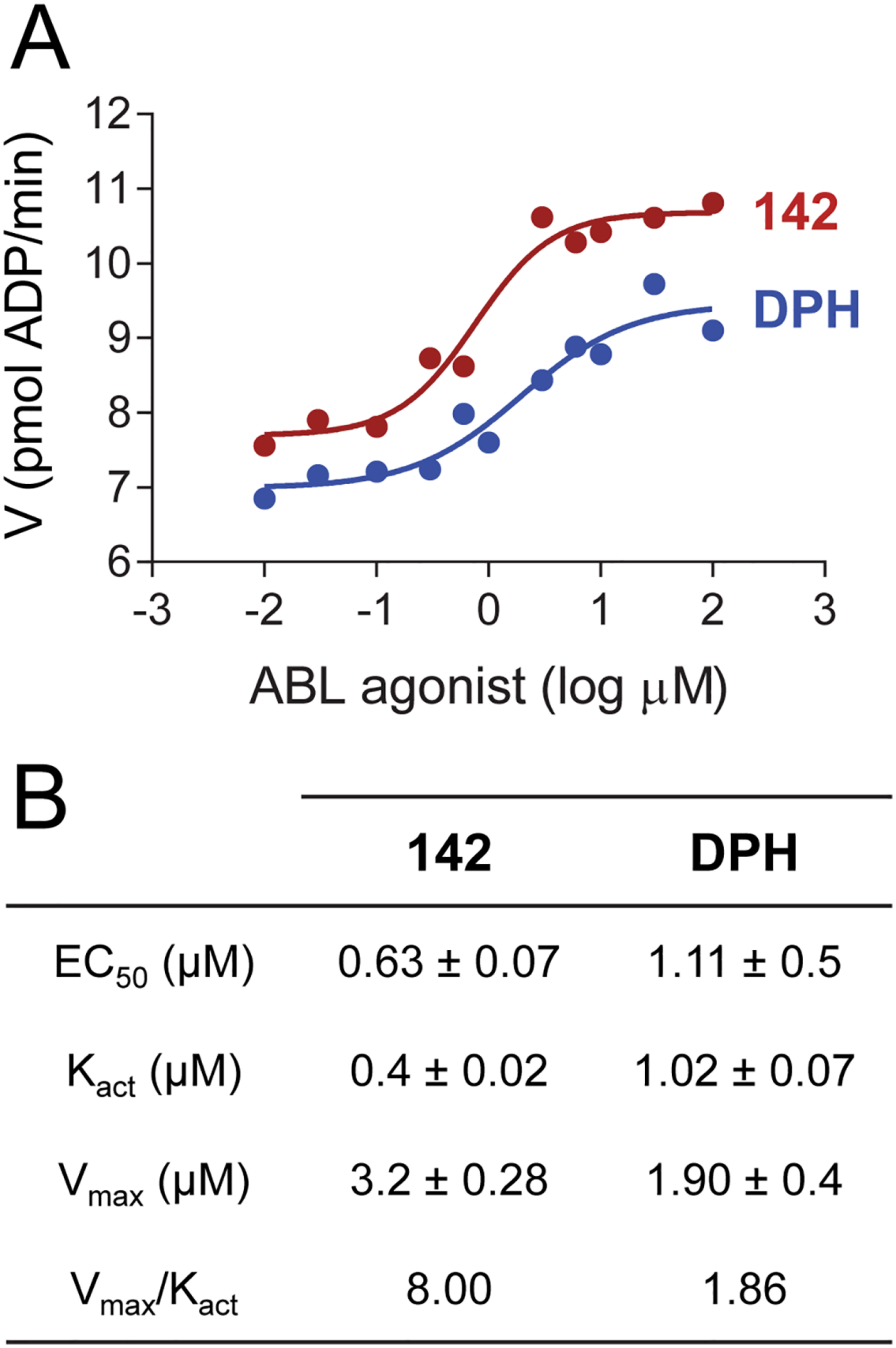
Concentration-dependent activation of the ABL kinase core protein by compound 142. The wild type ABL kinase core was assayed in the presence of compound 142 and DPH at the indicated concentrations using a kinetic kinase assay (see Materials and Methods). Reaction velocities are plotted as a function of compound concentrations. The resulting data were curve-fit to determine the EC_50_, K_act_ and V_max_ for each activator as described under Materials and Methods. Each of these parameters was determined in triplicate, and the mean values ± SE are presented in the table below the graph. The table also provides the ratio V_max_/K_act_ as a measure of overall enhancement of catalytic efficiency in the presence of each of these two ABL agonists.

To further characterize ABL activation by compound 142, we repeated kinetic kinase assays with the wild type ABL core over a range of compound concentrations. As shown in Fig 9, compound 142 activates the ABL core in a concentration-dependent manner, with an EC_50_ value of 0.63 ± 0.07 μM. This value compares favorably to that obtained with DPH, the myristic acid binding pocket agonist (EC_50_ = 1.11 ± 0.5 μM). We also calculated the activation constant (K_act_) for each compound from these data, which is defined as the concentration at which the reaction rate reaches half-maximum velocity (V_act_). For compound 142, the K_act_ was calculated as 0.4 ± 0.02 μM, while DPH yielded a value of 1.02 ± 0.07 μM. This analysis also showed that the extent of ABL activation by compound 142 was higher than that observed for DPH, based on the V_max_.

### Molecular dynamics simulations and docking studies predict binding of compound 142 to the SH3:linker interface in the ABL kinase core

**Fig 10:**
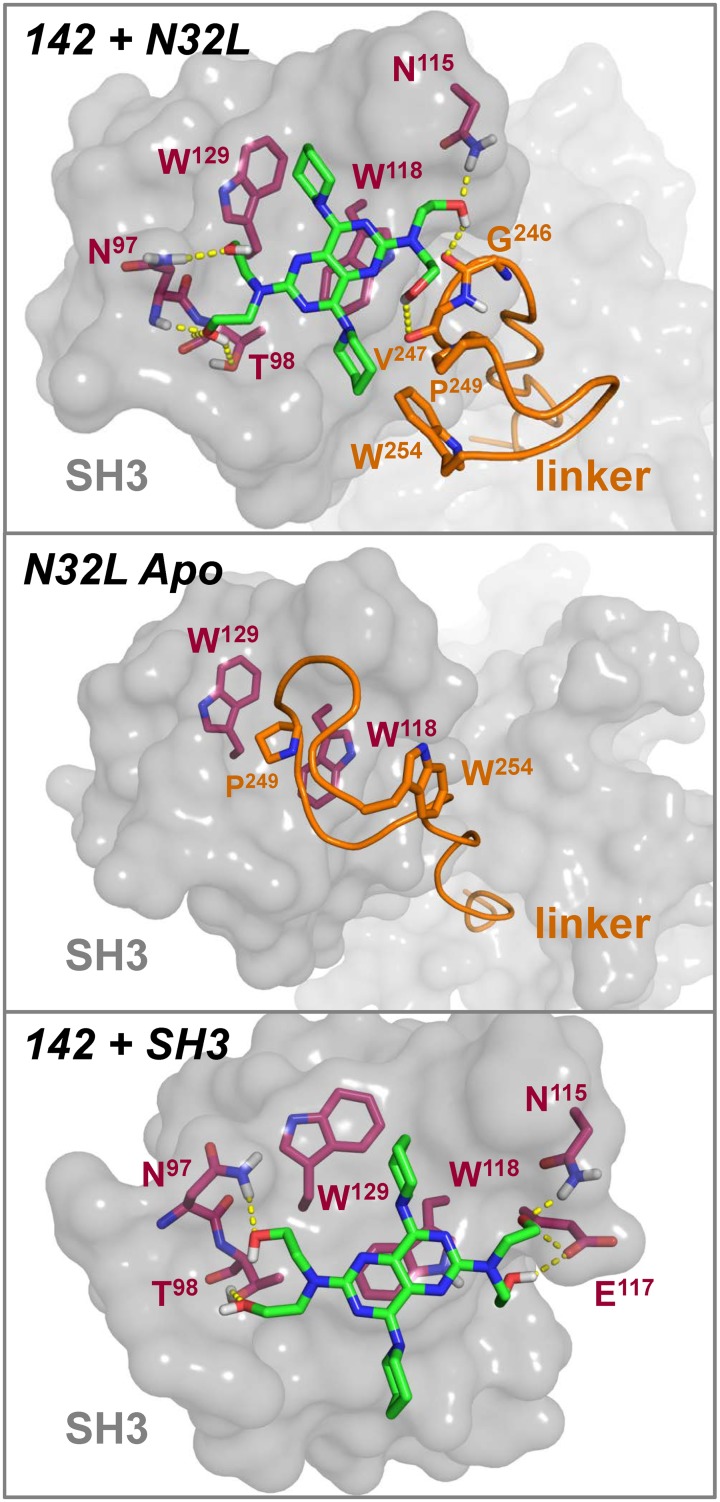
Molecular dynamics (MD) and molecular docking predict binding of compound 142 to the ABL SH3:linker interface. *Top*: The lowest energy pose of the ligand (compound 142; carbon atoms rendered in green) is shown docked to a snapshot of an MD simulation of the ABL N32L structure. SH3 domain residues predicted to contribute to ligand binding include Asn97, Thr98, Asn115, Trp118, and Trp129 (carbons in red). The backbone of the linker is shown as an orange ribbon, with Gly246, Val247, Pro249 and Trp254 predicted to contribute to the binding pocket. One of the piperidine groups of compound 142 makes hydrophobic contacts with linker Pro249 and Trp254, while the pyrimido-pyrimidine scaffold of compound 142 is π-stacking with Trp118. *Middle panel*: Model of the SH3:linker interface in the N32L region based on the crystal structure of the downregulated ABL core (PDB:2FO0), highlighting the interaction of linker Pro249 with SH3 Trp118 and Trp129. Ligand binding (top panel) is predicted to displace this regulatory interaction, leading to kinase activation. *Lower panel*: The lowest energy pose of compound 142 is shown docked to a snapshot of an MD simulation of the SH3 domain in the absence of the linker. The position of the 142 ligand is similar (within 1.5 Å RMSD) to that in the SH3 domain of N32L (top), except that the ligand contacts Glu117 rather than linker residues Gly246 and Val247. Without the linker, the potential hydrophobic stabilization of the 142 piperidine group is also lost.

Data presented in the previous sections demonstrate that compound 142 interacts with the regulatory N32L region of ABL, resulting in a decrease in thermal stability and a concomitant increase in kinase activity. We used molecular dynamics (MD) simulations to explore the dynamics of the N32L region used in the assays. To model the effect of the linker being displaced from the SH3 domain, we manually pulled the linker a short distance away from the SH3 domain prior to the simulation (see Materials and Methods). After approximately 20 ns, the linker reconnected with the SH3 domain through the interaction of linker Pro249 and SH3 Trp118. To explore possible binding sites for this compound on the N32L region of the ABL kinase core, we used the computational docking tool *smina* [32] to dock 142 to snapshots of the simulation prior to the reconnection of the SH3:linker interface. As shown in Fig 10 (top panel), compound 142 fits into a surface pocket defined by the SH3:linker interface in the N32L protein. This predicted binding site involves an aromatic interaction between the pyrimido-pyrimidine moiety of 142 and the indole side chain of SH3 Trp118, as well as polar contacts involving all four hydroxyl groups on the ligand. This aromatic interaction is consistent with probe peptide displacement as well as the observed decrease in the FP signal produced by the W118A mutation (Fig 5C). Two of the hydroxyl groups of 142 make potential hydrogen bonds with the side and main chains of SH3 Asn97 as well as the side chain of Thr98. The other two hydroxyl groups of 142 form hydrogen bonds with the main chain carbonyls of linker Gly246 and Val247 as well as the side chain of SH3 Asn115. In addition, one of the piperidine groups of compound 142 makes hydrophobic contacts with SH3 Trp129, while the other approaches the side chains of linker residues Pro249 and Trp254. Note that in the crystal structure of the fully assembled, downregulated conformation of the ABL core, linker Pro249 inserts between SH3 Trp118 and Trp129 (Fig 10, middle panel); displacement of this regulatory contact by compound 142 binding may contribute to kinase activation.

For comparison, we also ran unconstrained MD simulations of the SH3 domain in the absence of the linker. Our docked model to a snapshot from this simulation is shown in Fig 10 (bottom panel). The overall position of the ligand in this model is quite similar to that observed with the N32L snapshot (within 1.5 Å RMSD), and includes the potential stacking interaction with SH3 Trp118 and hydrogen bonding to SH3 Asn97, Thr98 and Asn115. Polar contacts of compound 142 with linker Gly246 and Val247 as well as hydrophobic interactions with linker Trp254 and Pro249 are not possible. However, additional hydrogen bonds are observed with SH3 Glu117. Loss of these hydrophobic interactions in the SH3-only model helps to explain the lower binding affinity of compound 142 for the isolated SH3 domain (K_D_ = 3.00 ± 0.40 x 10^−5^ M) relative to the N32L protein (K_D_ = 6.90 ± 0.78 x 10^−6^ M) as determined by SPR (data not shown and Fig 7B).

### Compound 142 does not activate the SRC-family kinase, HCK

**Fig 11:**
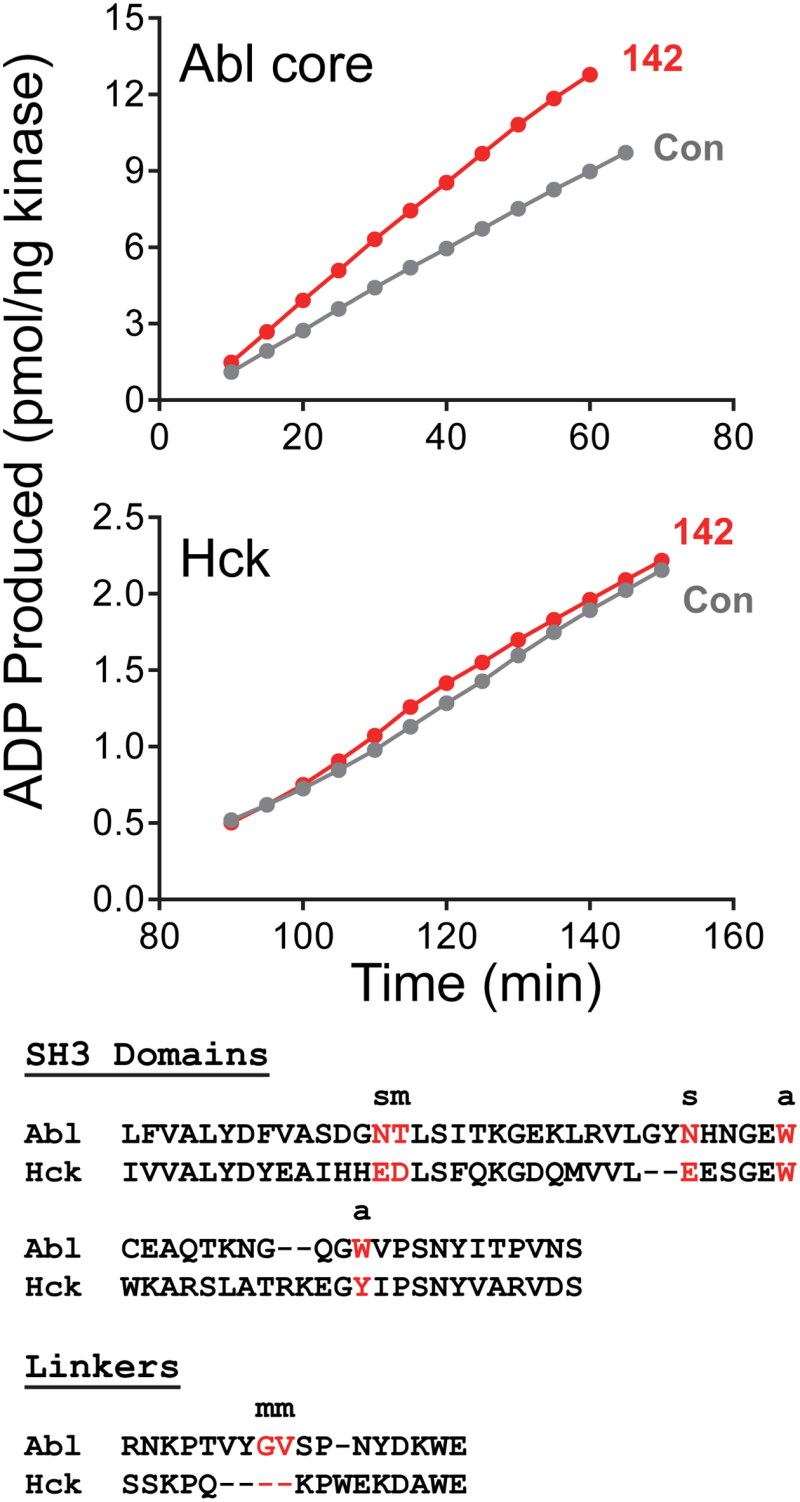
Compound 142 fails to activate downregulated HCK in vitro. *Top panels*: Recombinant, downregulated wild type ABL core and near full-length HCK proteins were assayed in the presence of compound 142 (1 μM) or with DMSO alone as control (Con) using a kinetic kinase assay (see Materials and Methods). Data are plotted as pmol ADP produced per ng kinase as a function of time. *Bottom*: Amino acid sequence alignment of the ABL and HCK SH3 domains and SH2-kinase linkers. SH3 and linker residues predicted to contribute to compound 142 binding are highlighted in red (see Fig 10). The type of interaction is indicated as side chain (s), main chain (m), or aromatic (a).

Like ABL, SRC-family kinases exhibit a very similar arrangement of SH3, SH2, and kinase domains in the downregulated conformation, and are also susceptible to activation by mutations and binding partners that disrupt intramolecular SH3:linker interaction [37,38]. To determine whether compound 142 also activates SRC-family kinases by a similar SH3:linker displacement mechanism, we performed kinetic kinase assays on the SRC-family member HCK in the presence and absence of this ABL activator. As shown in Fig 11, addition of compound 142 to recombinant near-full-length HCK had no effect the reaction velocity, suggesting that it is selective for ABL. Alignment of the SH3 domain and SH2-kinase linker sequences supports this view (Fig 11, bottom). Five of the seven residues predicted by the docking model to participate in compound 142 binding to the ABL SH3:linker surface are either substituted with different amino acids or are missing from the HCK sequence. These observations suggest that even subtle differences in the SH3 and linker sequences of ABL and SRC-family kinases can be exploited for the development of selective agonists or antagonists.

### Compound 142 cooperates with DPH to activate ABL in cells

**Fig 12:**
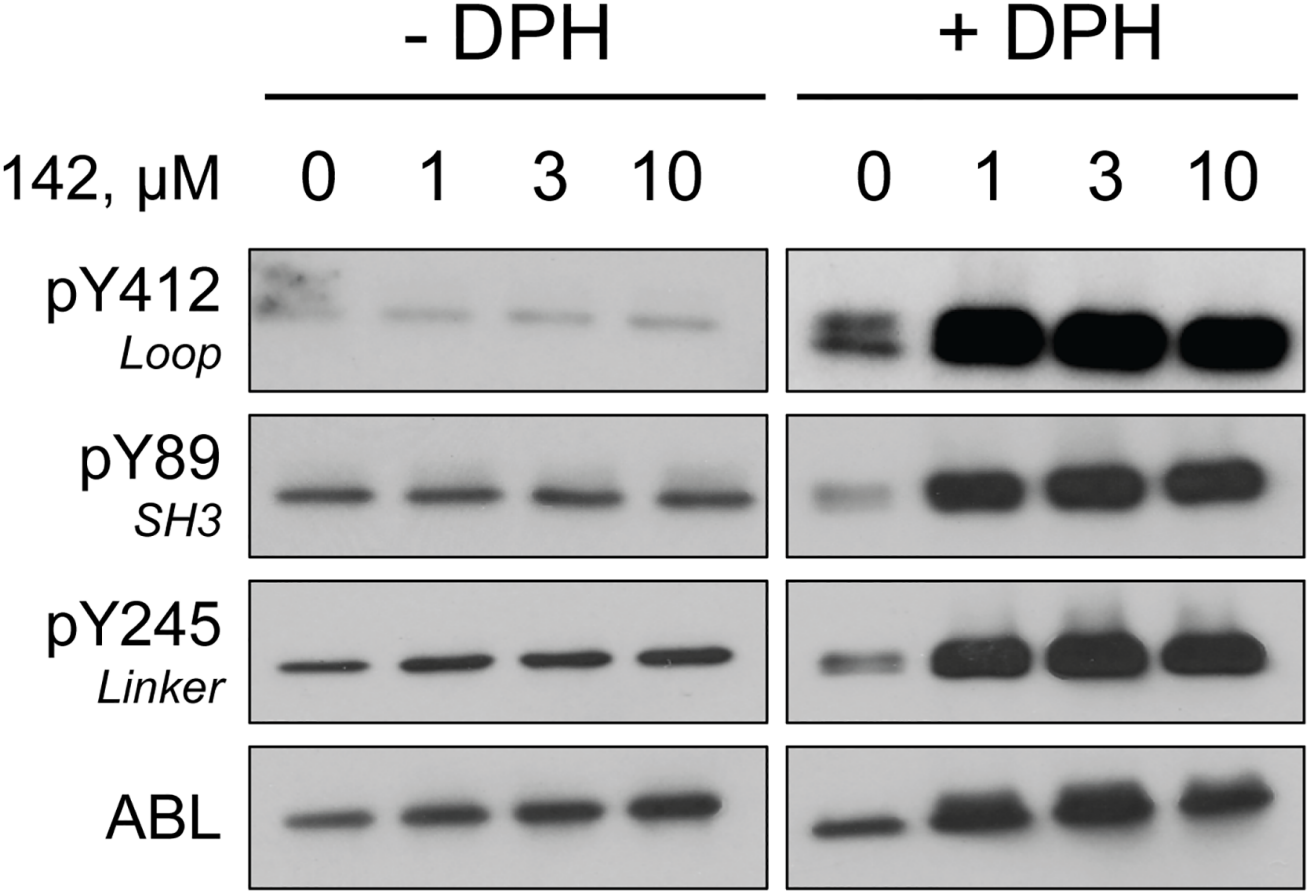
Compound 142 cooperates with DPH to stimulate ABL core autophosphorylation in cells. Human embryonic kidney 293T cells were transfected with an expression vector for the wild type ABL core followed by treatment with compound 142 overnight over the range of concentrations shown. The experiment was performed in the absence or presence of the myristic acid binding pocket agonist DPH at a final concentration of 10 μM. ABL proteins were immunoprecipitated from clarified cell lysates, and analyzed by immunoblotting with phosphospecific antibodies to three regulatory sites: pTyr412 in the activation loop, pTyr89 in the SH3 domain, and pTyr245 in the SH2-kinase linker, as well as an ABL antibody to control for protein recovery. This experiment was repeated twice with comparable results.

In a final series of studies, we evaluated the effect of compound 142 on the activity of the ABL core in cells. For these studies, we expressed the wild type ABL core in 293T cells, and then treated the cells over a range of compound 142 concentrations (1–10 μM). We then immunoprecipitated ABL and analyzed the level of phosphorylation at three important regulatory tyrosine sites by immunoblotting with phosphospecific antibodies [8,39]: pY412 (activation loop), pY245 (SH2-kinase linker), and pY89 (SH3 domain). As shown in Fig 12, treatment with compound 142 alone did not affect phosphorylation of these sites. We then repeated this experiment in the presence of DPH, the myristic acid binding pocket activator described above, at a concentration of 10 μM. In the absence of compound 142, DPH had a very small effect on ABL phosphorylation. However, the combination of compound 142 and DPH led to remarkable enhancement of phosphorylation on all three regulatory tyrosines, providing strong evidence for an activating effect of compound 142 on ABL in cells. These observations suggest that in the cellular environment, unlike in vitro, compound 142 alone may be not sufficient to disturb SH3:linker interaction and stimulate ABL activity. However, when the regulatory interaction of the myristoylated Ncap with the kinase domain C-lobe is perturbed by DPH, then the activating effect of SH3:linker displacement becomes readily apparent.

## Summary and Conclusions

In this study, we developed a screening strategy to identify allosteric small molecule modulators of ABL kinase activity that work outside of the kinase domain. Our FP-based assay targets the regulatory domains of ABL that control its kinase activity through intramolecular interactions. Specifically, this assay is based on a recombinant ABL protein comprising the complete regulatory apparatus (Ncap-SH3-SH2-linker) and a synthetic polyproline probe peptide (p41) that selectively binds the ABL SH3 domain. Interaction of the probe peptide with the ABL N32L protein results in a robust and reproducible FP signal. Mutation of the SH3 binding site (W118A) or introduction of a high-affinity linker both resulted in loss of the FP signal, demonstrating that probe access requires an intact and accessible SH3 domain. A small-scale pilot screen of 1200 FDA-approved compounds identified dipyridamole (compound 142) as an inhibitor of the FP signal observed with the N32L:p41 complex, and direct interaction of this compound with the ABL N32L protein was confirmed by SPR and DSF assays. Dipyridamole was observed to stimulate the kinase activity of downregulated ABL kinase in vitro, and was more potent than the previously described ABL agonist DPH which targets the myristic acid binding pocket in the kinase domain. In contrast to wild type ABL, dipyridamole had no effect on a modified ABL core protein with a high-affinity linker, suggesting that it works by binding to the SH3 domain and disrupting the SH3:linker interface. Molecular dynamics simulations in combination with molecular docking support this proposed mechanism of action: dipyridamole was predicted to interact with the ABL core through a pocket defined by the SH3:linker interface. This interaction involved Trp118 on the SH3 domain binding surface, potentially disrupting a key regulatory interaction with Pro249 in the linker. Discovery of dipyridamole as an ABL agonist provides an important proof of concept that small molecules altering SH3:linker interaction represent allosteric modulators of ABL kinase activity. Selective agonists of ABL function have potential as chemical probes to better understand the role of ABL kinase activity in solid tumors and in response to genotoxic stress. Conversely, allosteric antagonists may also be discovered by this approach and have the potential to complement current ATP-competitive inhibitors of BCR-ABL in the context of CML and other cancers. The allosteric inhibitor discovery concept may also be extended to the discovery of allosteric modulators of other kinases systems with multi-domain regulatory interactions, including members of the SRC and TEC kinase families.
